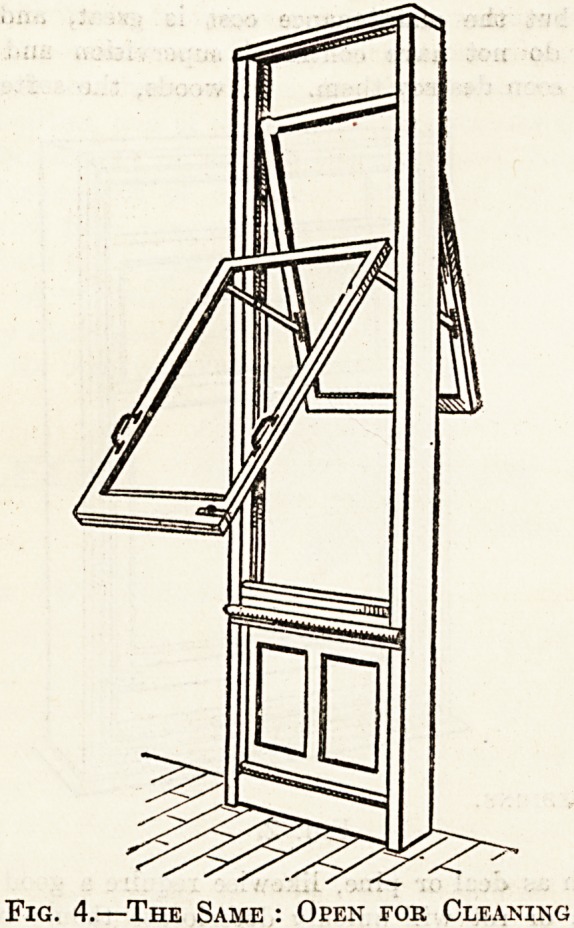# The Latest Types of the Ward Window

**Published:** 1912-04-13

**Authors:** 


### HOSPITAL ARCHITECTURE AND CONSTRUCTION.

[Communications on this subject should be marked "Architecture" in the left-hand top corner of the envelope.]

### The Latest Types of the Ward Window.

The ward window is in a transition stage, for with the birth of reinforced and steel skeleton buildings it would be possible to make the walls all window. Light is an essential in the cure of disease, but as glass is more conductive than a solid wall, it is not desirable to make the walls all glass.

Miss Nightingale advocated that one-third of the wall area should be of glass; no doubt structural considerations caused the lowness of her computation, for building methods have advanced by rapid strides since her day. As she so aptly remarked, "We can generate warmth; we cannot generate daylight or the purifying and curative effect of the sun's rays."

Bronze is without question the ideal material of which to form the framing, for two especial reasons: first, the maintenance cost is nil; and secondly, the area taken up by the framing is considerably less than in the case of wood. Iron would take up the same area as bronze approximately; but the maintenance cost is great, and if the windows do not have continual supervision and repair, rust will soon destroy them. Of woods, the softer varieties, such as deal or pine, likewise require a good deal of painting, or rot will quickly deteriorate them; but with the hard woods, such as oak or teak, the only attention required is an occasional oiling. It may be argued that teak or oak internally looks rather dead unless enamelled, and this is undoubtedly so, but experience goes to prove that it is in hospital work too frequently the external dilapidations which are neglected, and not the internal painting.

The highest authorities seem to favour that style of window which consists of a double-hung sash with a hopper fanlight over it. The fanlight is hinged at the bottom and falls inwards on a quadrant, and the side

Fig. 1. Mr. Gibbon's Two Designs. Fig. 2.

Fig. 3.—The Lever Window: Open and Shut.

spandrels are glazed to avoid down draughts on the patients. The cleaning of the windows in a large establishment is a matter requiring some little consideration; a typical form of contract for a large London institution provides that certain windows are to be cleaned once in two weeks and the remainder (including wards) once in four weeks. This means that in the higher storeys a man has to get through the sash window and stand on the sill, possibly some fifty feet above the ground. The risk to life is great, and must in some manner of insurance be paid for by the institution; and the act of getting outside the window means at the end of the year a considerable outlay for making good to decoration and painted work. Probably with these points in view, several enterprising firms have devised types of sash which can be cleaned on both sides from inside. In general principle these consist of a sash pivoted in or near the centre of its vertical height on each side, and it can be folded over so that a window-cleaner can reach its whole area. Our illustrations (figs. 1 and 2) show a type of this window, taken from a model by Mr. James Gibbons, of Wolverhampton. It consists, as before stated, of sashes pivoted near the centre, and in ordinary use is held in position by thumb-screws so that it acts as an ordinary double-hung sash. The cost of the metal fittings for this type of window is about half a sovereign, and to this must be added a few shillings for extra joiner's work. Hemp sash-cords have only a limited life; this difficulty may be overcome by hanging the sashes on chains like a cycle-chain. Probably this is one of the factors which caused Mr. Keith Young to use an ingenious type of window at the Miller General Hospital, Greenwich, in which there are neither chains, counterbalance weights, nor running wheels. We believe Mr. W. A. Pite is using the same type of window at Denmark Hill. Our illustration (fig. 3) shows a ward where the window is seen both open and closed. The principle on which it is based is that of the lever, a simple form that need not entail complications in design: by opening the lower sash the upper one is correspondingly opened and held in position, the opposites being in equilibrium. Fig. 4 shows how this type of window is opened inwards so that it can be easily cleaned, and it is interesting to note that window-cleaning companies offer a rebate of 50 per cent. from their usual charges for buildings fitted with this sash. So many eminent architects favour this patent fitting that we can confidently advise any who contemplate extensions to their premises to inquire into its relative merits. It is made by the Austral Window-Balance Company, whose London offices are at No. 3 Victoria Street, S.W.

Fig. 4.—The Same: Open for Cleaning

## Figures and Tables

**Fig. 1. Fig. 2. f1:**
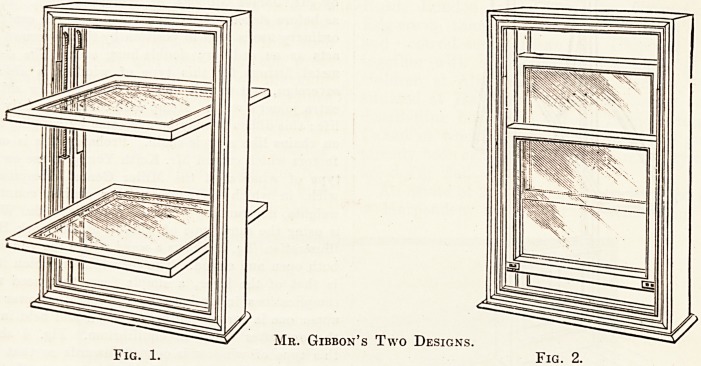


**Fig. 3. f2:**
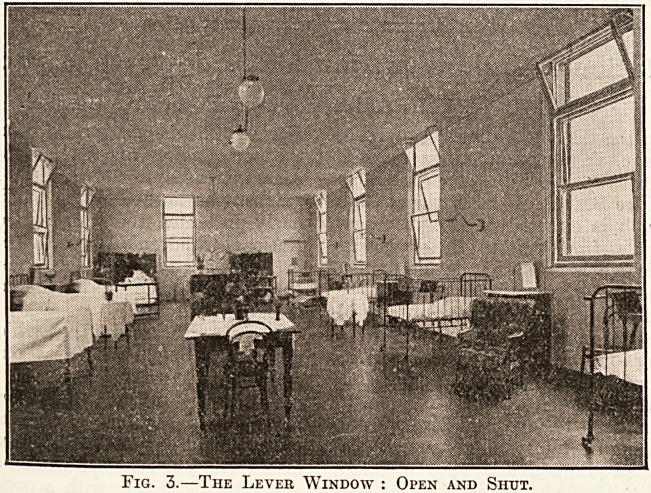


**Fig. 4. f3:**